# Contrast-Enhanced Spectral Mammography-Based Radiomics Nomogram for the Prediction of Neoadjuvant Chemotherapy-Insensitive Breast Cancers

**DOI:** 10.3389/fonc.2021.605230

**Published:** 2021-02-22

**Authors:** Zhongyi Wang, Fan Lin, Heng Ma, Yinghong Shi, Jianjun Dong, Ping Yang, Kun Zhang, Na Guo, Ran Zhang, Jingjing Cui, Shaofeng Duan, Ning Mao, Haizhu Xie

**Affiliations:** ^1^ School of Medical Imaging, Binzhou Medical University, Yantai, China; ^2^ Department of Radiology, Yantai Yuhuangding Hospital, Affiliated Hospital of Qingdao University, Yantai, China; ^3^ Department of Pathology, Yantai Yuhuangding Hospital, Affiliated Hospital of Qingdao University, Yantai, China; ^4^ Department of Breast Surgery, Yantai Yuhuangding Hospital, Affiliated Hospital of Qingdao University, Yantai, China; ^5^ Collaboration Department, Huiying Medical Technology Co., Ltd, Beijing, China; ^6^ Precision Health Institution, GE Healthcare, Shanghai, China

**Keywords:** radiomics, breast cancer, contrast-enhanced spectral mammograph, neoadjuvant chemotherapy, oncology

## Abstract

**Purpose:**

We developed and validated a contrast-enhanced spectral mammography (CESM)-based radiomics nomogram to predict neoadjuvant chemotherapy (NAC)-insensitive breast cancers prior to treatment.

**Methods:**

We enrolled 117 patients with breast cancer who underwent CESM examination and NAC treatment from July 2017 to April 2019. The patients were grouped randomly into a training set (n = 97) and a validation set (n = 20) in a ratio of 8:2. 792 radiomics features were extracted from CESM images including low-energy and recombined images for each patient. Optimal radiomics features were selected by using analysis of variance (ANOVA) and least absolute shrinkage and selection operator (LASSO) regression with 10-fold cross-validation, to develop a radiomics score in the training set. A radiomics nomogram incorporating the radiomics score and independent clinical risk factors was then developed using multivariate logistic regression analysis. With regard to discrimination and clinical usefulness, radiomics nomogram was evaluated using the area under the receiver operator characteristic (ROC) curve (AUC) and decision curve analysis (DCA).

**Results:**

The radiomics nomogram that incorporates 11 radiomics features and 3 independent clinical risk factors, including Ki-67 index, background parenchymal enhancement (BPE) and human epidermal growth factor receptor-2 (HER-2) status, showed an encouraging discrimination power with AUCs of 0.877 [95% confidence interval (CI) 0.816 to 0.924] and 0.81 (95% CI 0.575 to 0.948) in the training and validation sets, respectively. DCA revealed the increased clinical usefulness of this nomogram.

**Conclusion:**

The proposed radiomics nomogram that integrates CESM-derived radiomics features and clinical parameters showed potential feasibility for predicting NAC-insensitive breast cancers.

## Introduction

Neoadjuvant chemotherapy (NAC) represents the primary and direct treatment modality of locally advanced breast cancers (1). The main advantages of NAC treatment are the reduction of tumor volume and metastasis, increased breast-conserving surgery probabilities instead of mastectomy, and determination of drug sensitivity (2–4). Nevertheless, response rates to NAC vary among patients due to intrinsic heterogeneity influenced by molecular features, clinical behavior, and morphological appearance (5). Approximately 10–35% of patients may be insensitive to NAC, and 5% have further disease progression after NAC (6). In these patients, NAC is proportionally less beneficial, delays surgery, contributes to poor prognosis, and increases treatment costs. Thus, an accurate method for predicting treatment resistance prior to NAC is necessary for personalized clinical strategies and further optimal triage of care, especially for short term survivors.

Varying methods are used to evaluate the response to NAC. Among them, magnetic resonance imaging (MRI) is regarded as the “gold-standard” for assessing response to NAC (7). when MRI is contraindicated, contrast-enhanced spectral mammography (CESM) may be a substitute novel instrumentation for breast cancer diagnosis, as revealed in recent studies (8). CESM has better sensitivity (SEN), significantly shorter exam time, and lower cost and higher negative predictive value (NPV), positive predictive value (PPV)and lesser background enhancement than MRI (9–12). Patients exhibit significantly high overall preference toward CESM, due to great comfort and low rate of anxiety (13).

Initial work on assessing treatment response was focused on the imaging measurements of tumor diameter before NAC, after NAC, and sometimes during NAC according to the response evaluation criteria in solid tumor (RECIST) criteria (14, 15). Despite the merits of CESM examination, the changes in tumor size on the image occurring after NAC treatment limits the role of CESM in the early determination of therapeutic outcomes. As a new method, radiomics has currently gained recognition in the field of oncology for noninvasive analysis (16). More specifically, previous studies revealed that response to anti-tumor therapy can be assessed using radiomics analysis, exemplified by rectal and breast cancers (6, 17–19). Moreover, our group has also achieved some encouraging outcomes in the field of breast cancer based on radiomics methods (20–22). Radiomics involves extracting quantitative imaging features to investigate associations between radiomics feature and clinicopathology beyond human capabilities (23–25) and connects medical imaging and precision medicine (26). A wide cluster of machine learning methods, including logistic regression analysis, random forest and support vector machine, have been successfully applied to various clinical research areas.

In this preliminary research, we developed and validated a radiomics nomogram based on CESM-derived radiomics features and clinical risk factors for the pretreatment determination of NAC-insensitive breast cancers.

## Materials and Methods

### Patients

This study was reviewed and approved by the Research Ethics Committee of Yantai Yuhuangding Hospital, and patient informed consent was waived. The initial cohort of 198 patients was retrospectively reviewed through July 2017 to April 2019. All eligible patients met the following inclusion criteria: (i) biopsy-confirmed unilateral invasive breast cancer without distant metastasis; (ii) no prior treatment other than NAC and no history of other malignancy; (iii) CESM examination conducted before and after the initiation of NAC; and (iv) a pathologic examination performed before the implementation of NAC. The exclusion criteria were as follows: (i) multifocal or bilateral lesions (ii) lack of CESM image data or clinical data before and after NAC; (iii) surgery not performed or incomplete immunohistochemical information; and (iv) insufficient CESM image quality for measurements. The entire cohort of 117 patients conforming to the inclusion criteria was divided randomly into the training and validation sets in a ratio of 8:2. Correspondingly, different treatment regimens were also randomly distributed in the training and validation sets.

### CESM Data Acquisition

Only the CESM images before initiating NAC with cranial caudal (CC) projection of the eligible patients were routinely acquired from our Department of Radiology in this study, mainly including the low-energy and recombined images of suspected side in DICOM format. CESM is based on a dual-energy system developed by GE Healthcare (Chalfont St-Giles, UK): following 2 min of injection of an iodinated contrast agent (1.5 ml/kg body weight), and low- and high-energy images are acquired in rapid succession while the breast remains compressed, from which a recombined image is obtained. The average gland dose of examination is 3mGy. The low-energy image is the same as a conventional mammogram, whereas a recombined image shows contrast medium uptake (27–29). In a monolateral CESM, the radiographer compressed the breast for the mediolateral oblique (MLO) view 2 min after injecting the contrast agent and then decompressed the breast, and the breast was compressed for the CC view after another 2 min (14).

### NAC Scheme and Response Assessment

All patients received NAC treatment before breast surgery, following the National Comprehensive Cancer Network (NCCN) guideline (1). 98 patients (84%) received taxane-based NAC schemes and 19 patients (16%) received anthracycline- and taxane-based NAC schemes. Furthermore, human epidermal growth factor receptor-2 (HER2) positive patients also received trastuzumab.

RECIST criteria was used to assess the response to NAC by comparing the largest dimension of tumor at baseline (pre-NAC) with that of residual lesion later during treatment (post-NAC) on the recombined image with CC projection. According to the reference standard, patients with tumors graded as stable disease (SD, < 30% dimensional reduction/< 20% dimensional increase) or progressive disease (PD ≥ 20% dimensional increase) were included in the NAC-ineffective group.

### Tumor Masking and Radiomics Feature Extraction

**Figure 1:**
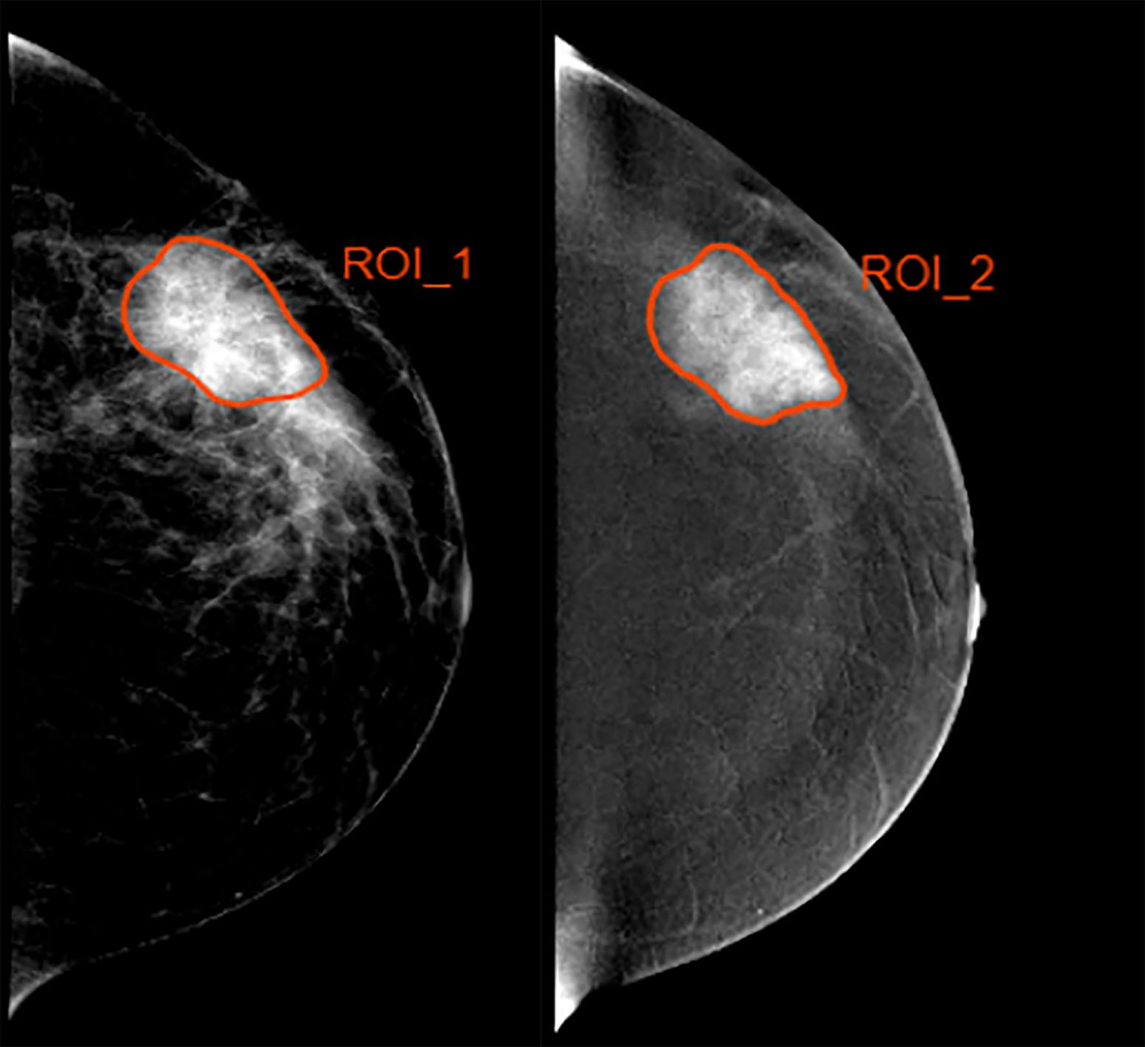
Example of delineating region of interest (ROI) in a 35 year-old woman with a 4.5-cm mass in the left breast. (Left) The low-energy and (Right) recombined images with cranial caudal (CC) projection.

Two experienced breast radiologists blinded to pathological outcomes manually delineated the tumor region of interest (ROI) by outlining the tumor margin on the low-energy and recombined images with standard CC projection before NAC *via* the ITK-SNAP software, as shown in **Figure 1**. If contradictory, other senior radiologists will evaluate the tumor mask again to reach agreement. The recombined images were used as reference to determine the tumor boundary on the low-energy images. Radiomics features per patient were then extracted from pretreatment CESM images with manually segmented ROIs. The task of radiomics feature extraction was conducted in the AK software (Artificial Intelligence Kit; GE Healthcare, China, Shanghai).

To ensure reproducibility of radiomics feature extraction, we employed intra-class correlation coefficients (ICCs) for assessing the intra- and inter-observer agreement of ROI delineation. First, two radiologists with 8 years (Reader 1) and 9 years (Reader 2) of experience in diagnosis of breast cancer delineated the ROI of 30 randomly chosen CESM images. One week later, Reader 1 repeated the same procedure. An ICC > 0.75 was considered as substantial agreement.

### Radiomics Feature Selection and Radiomics Score Construction

Before the selection of radiomics feature, the normalization processing of all extracted features was performed in the training set, followed by sample augmentation technology which was used to artificially increase the training data set. First, features with ICC >0.75 within the training set were retained. Second, features were further selected using analysis of variance (ANOVA). Third, we applied the least absolute shrinkage and selection operator (LASSO) regression for selecting the key radiomics features with nonzero coefficients, and a 10-fold cross-validation with a maximum area under the receiver operator characteristic (ROC) curve (AUC) criterion was conducted to determine an optimal regulation weight (λ). A radiomics score for each patient was then computed using a linear combination of the key features weighted by their LASSO coefficients. We finally calculated AUC value to assess the predictive performance of the radiomics score.

### Clinical Factor Selection, and Clinical Model Construction

Pretreatment clinicopathologic information was collected and identified by two experienced radiologists. ANOVA was applied to clinical variables for selecting the optimal clinical parameters. The clinical model was then developed with the key clinical risk factors by using multivariate logistic regression analysis.

### Construction and Assessment of Radiomics Nomogram

A radiomics nomogram incorporating radiomics score and clinical risk factors was built using the multivariate logistic regression in the training set and used as a convenient visible tool to predict the individual probability of NAC-insensitivity.

The discrimination power of the radiomics nomogram was quantified by calculating AUC in both sets. In addition, the point on the ROC curve farthest from the diagonal line corresponds with the largest of the Youden index by calculating the sum of SEN and specificity (SPE) and then subtracting 1 over all possible threshold values, which was used to determine the cutoff value dividing the NAC-ineffective and NAC-effective patient predictive values. According to the cutoff value, the accuracy, PPV, NPV, SEN, and SPE were calculated in both sets.

Decision curve analysis (DCA) was employed to evaluate the benefit of nomogram-assisted decisions in a clinical context. The net benefit was calculated by subtracting the proportion of all false positive patients from the proportion of true positive patients. Standardized net benefit was scaled into the range between 0 and 1.

### Statistical Analysis

The radiomics nomogram was developed in the training set by using multivariate logistic regression, whereas the validation set was used to validate the radiomics nomogram. Clinical characteristics between two sets were compared using the chi-squared or fisher exact tests for categorical variables and independent sample t test for continuous variables. The DeLong test was used to determine the statistical significance of the AUC of different models. LASSO regression analysis was performed by “python” scikit-learning package, and ANOVA was performed with the “anova” software package. ROC curves were plotted with the “roc” software package, and DCA was performed with the “decision-curve” software package. The statistical analyses were conducted using the R software version 3.5.3. P values < 0.05 were interpreted as statistical significance.

## Results

### Clinical Characteristics

**Figure 2:**
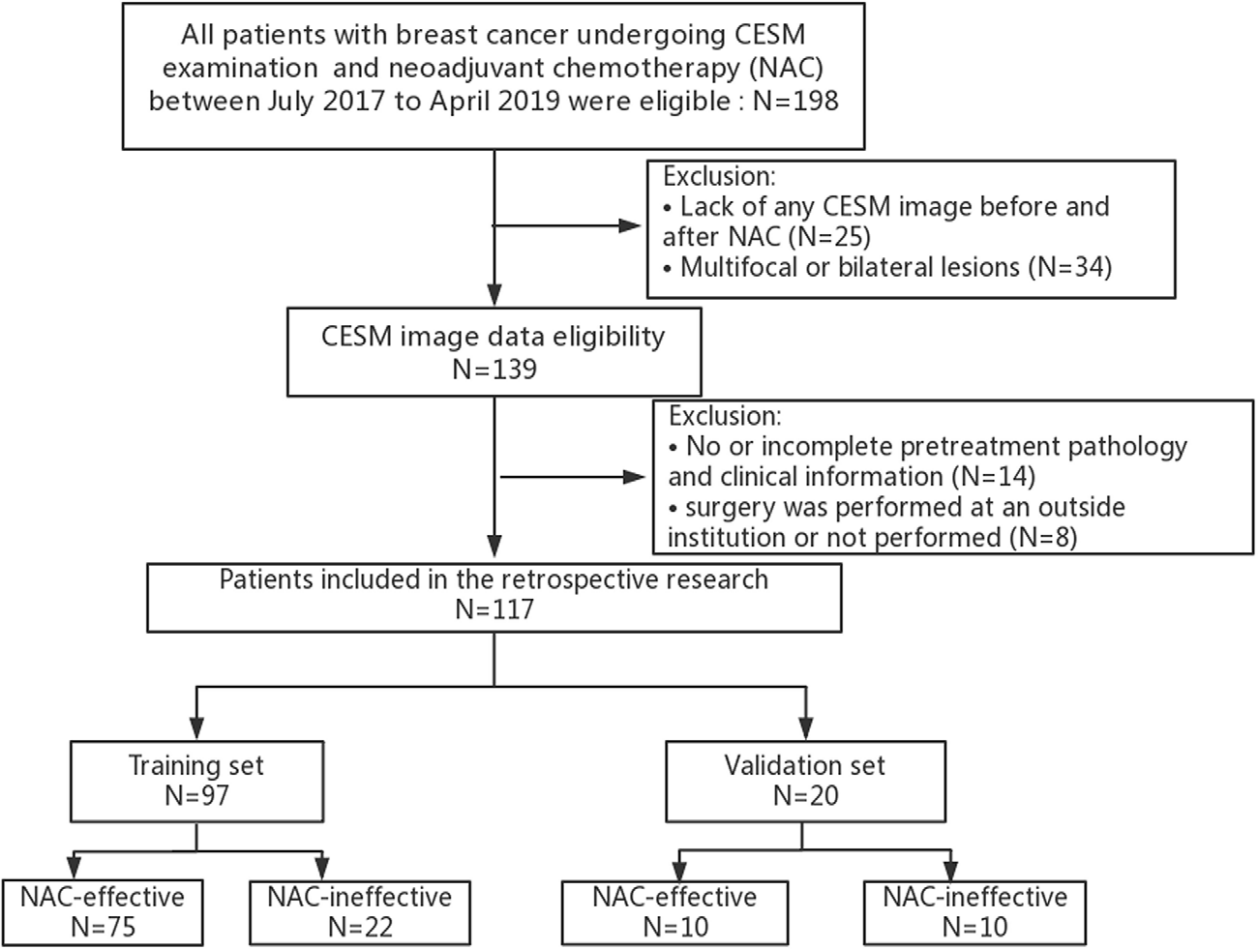
Flow chart of the study population with exclusion criteria.

A total of 198 patients with breast cancer undergoing NAC treatment were recruited in the study, and 117 patients were finally enrolled. The training set included 97 patients and the validation set included 20 patients, as shown in **Figure 2**. Patient Characteristics are shown in **Table 1**. No significant difference was found between the two sets in terms of no response prevalence (P = 22.7% and 50% in the training and validation sets, respectively, p = 0.82). No significant statistical differences in clinical characteristics were found between the NAC-effective and NAC-ineffective groups in the training set (P = 0.075–1), as well as in the validation set (P = 0.303–0.937).

**Table 1:** Clinical characteristics in the training and validation cohorts.

Characteristics	Training set (N = 97)		p	Validation set (N = 20)		p
	NAC-ineffective(N = 22)	NAC-effective(N = 75)		NAC-ineffective(N = 10)	NAC-effective(N = 10)	
Age(years), (mean ± SD), years	48.7 ± 10.2	49.5 ± 8.3	0.71	51.1 ± 7.0	49.3 ± 8.6	0.63
Size (cm), (mean ± SD), cm	5.2 ± 2.5	4.9 ± 2.8	0.73	4.9 ± 2.8	4.7 ± 3.2	0.88
ER status			0.17			0.58
Positive (N = 84)	18 (81.8%)	50 (66.7%)		9 (90.0%)	7 (70.0%)	
Negative (N = 33)	4 (18.2%)	25 (33.3%)		1 (10.0%)	3 (30.0%)	
PR status			0.34			0.65
Positive (N = 79)	17 (77.3%)	50 (66.7%)		7 (70.0%)	5 (50.0%)	
Negative (N = 38)	5 (22.7%)	25 (33.3%)		3 (30.0%)	5 50.0%)	
HER status			0.38			0.30
Positive (N = 49)	16 (72.7%)	28 (37.3%)		1 (10.0%)	4 (40.0%)	
Negative (N = 58)	6 (27.3%)	47 (62.7%)		9 (90.0%)	6 (60.0%)	
Ki67			0.07			1.00
Positive (N = 110)	19 (86.4%)	73 (97.3%)		9 (90.0%)	9 (90.0%)	
Negative (N = 7)	3 (13.6%)	2 (2.6%)		1 (10.0%)	1 (10.0%)	
Molecular subtype			0.60			0.45
Luminal (N = 88)	18 (81.8%)	54 (72.0%)		9 (90.0%)	7 (70.0%)	
HER2 over-expression (N = 16)	2 (9.1%)	13 (17.3%)		0 (0%)	1 (10.0%)	
Basal-like (N = 13)	2 (9.1%)	8 (10.7%)		1 (10.0%)	2 (20.0%)	
T stage			0.46			0.16
1 (N = 6)	0 (0%)	3 (4.0%)		0 (0%)	3 (30.0%)	
2 (N = 61)	14 (63.6%)	39 (52%)		5 (50.0%)	3 (30.0%)	
3 (N = 50)	8 (36.4%)	33 (44%)		5 (50.0%)	4 (40.0%)	
LNM			1.00			—
Positive (N = 115)	22 (100.0%)	73 (97.3%)		10(100.0%) (100.0%) (100.0%) (100.0%)	10(100.0%)	
Negative (N = 2)	0 (0%)	2 (2.7%)		0 (0%)	0(0%)	
BPE			0.53			0.18
Minimal (N = 60)	10 (45.5%)	41 (54.7%)		6 (60.0%)	3 (30.0%)	
Mild (N = 39)	7 (31.8%)	24 (32.0%)		2 (20.0%)	6 (60.0%)	
Moderate (N = 18)	5 (22.7%)	10 (13.3%)		2 (20.0%)	1 (10.0%)	
Marked (N = 0)	0 (0%)	0 (0%)		0 (0%)	0 (0%)	
BD			0.53			0.83
Almost fatty (N = 32)	7 (31.8%)	18 (24.0%)		4 (40.0%)	3 (30.0%)	
Scattered fibroglandular (N = 38)	7 (31.8%)	26 (34.7%)		2 (20.0%)	3 (30.0%)	
Heterogeneously (N = 36)	5 (22.7%)	26 (34.7%)		2 (20.0%)	3 (30.0%)	
Extremely dense (N = 11)	3 (13.6%)	5 (6.6%)		2 (20.0%)	1 (10.0%)	
NAC scheme			0.83			1.00
Taxane based (N = 98)	20 (90.9%)	67 (89.3%)		6 (60%)	5 (50%)	
Anthracycline and taxane	2(9.1%)	8(10.7%)		4 (40%)	5 (50%)	
based (N = 19)						

ER, estrogen receptor; PR, progesterone receptor; HER2, human epidermal growth factor receptor 2; LNM, lymph node metastasis; BPE, background parenchymal enhancement; BD, breast density; SD, standard deviation; NAC, neoadjuvant chemotherapy.

### Radiomics Feature Selection and Radiomics Score Development

**Figure 3:**
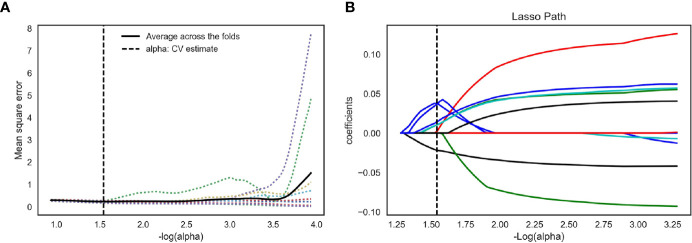
Feature selection for the LASSO logistic regression and the predictive accuracy of the radiomics signature. **(A)** Tuning parameter (λ) selection by 10-fold cross-validation with minimum criteria. Mean square error (y-axis) was plotted as a function of log(λ) (x-axis). The dotted vertical lines were drawn at the optimal value of λ, where the model provided its best fit of the data. The optimal value -log(alpha) = 1.50 **(B)** LASSO coefficient profiles for the whole features. The dotted vertical line was plotted at the value selected with 10-fold cross-validation, where eleven optimal features with nonzero coefficients are indicated in the plot.

**Figure 4:**
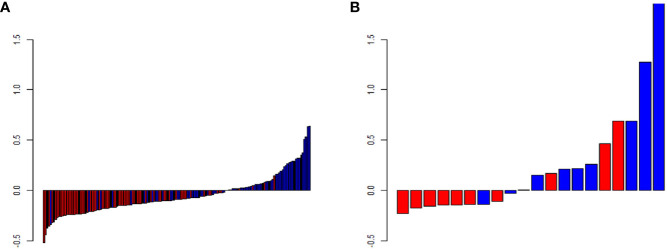
Predicted scores of patients in **(A)** the training and **(B)** validation set. The red color indicates NAC-ineffective patient and blue color indicates NAC-effective patient. The bars above the horizontal line indicates NAC-effective patient and the bars below the horizontal line indicates NAC-ineffective distinguished by the radiomics score.

We extracted 792 radiomics features from CESM images with manually segmented ROIs in the training set, including shape-and size-based, first-order statistical, and texture features. The substantial reproducibility of radiomics feature extraction were achieved with the intra- and inter- observer ICCs from 0.823 to 0.890 and 0.789 to 0.835, respectively. In order to determine the optimal regulation weight λ [−log(λ) = 1.5] for the LASSO algorithm, we finally screened 11 optimal radiomics features with nonzero coefficients for calculating radiomics score (**Figures 3A, B**, **Table 2**). The radiomics scores for each patient are shown in **Figure 4**. The red and blue colors indicate the NAC- ineffective and NAC-effective patient, respectively. The bars above and below the horizontal line indicate NAC-effective and NAC- ineffective patient as distinguished by the radiomics score, respectively. The results revealed that the radiomics score has a good predictive ability.

**Table 2:** Radiomics feature for establishing radiomics score.

Intercept and variable	Modality	Coefficient
Skewness	Low-energy image	−0.05380741
LongRunEmphasis_angle0_offset1	low-energy image	0.093650085
ShortRunEmphasis_angle135_offset7	Recombined image	−0.011611473
Inverse Difference Moment	Recombined image	0.006785827
RunLengthNonuniformity_AllDirection_offset1_SD	Recombined image	0.012658414
kurtosis	Recombined image	0.023503887
ShortRunEmphasis_AllDirection_offset1_SD	Recombined image	0.024338342
Large Area Emphasis	Recombined image	0.05187876
GreyLevelNonuniformity_AllDirection_offset1_SD	Recombined image	0.090814997
ShortRunLowGreyLevelEmphasis_angle0_offset4	Recombined image	0.105234074
GreyLevelNonuniformity_angle0_offset4	Recombined image	0.142534338

### Construction of Radiomics Nomogram

**Figure 5:**
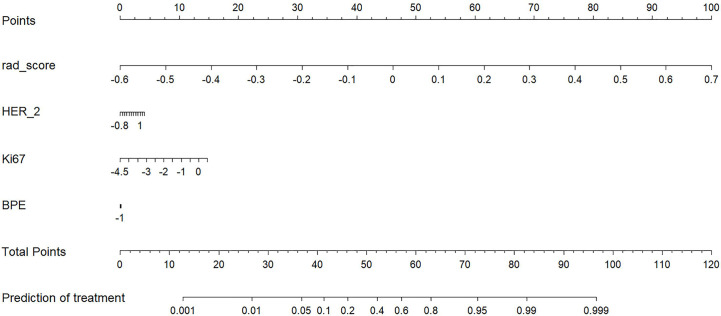
A radiomics nomogram for the prediction of NAC- ineffective patients in the primary cohort.

The radiomics score and clinical characteristics, namely, HER-2 status, Ki67 index, and background parenchymal enhancement (BPE), significantly predicted early NAC-ineffective patients. Thus, the radiomics nomogram was developed with the combination of radiomics score, HER-2 status, Ki67 index, and BPE as listed in **Figure 5**.

### Performances of Radiomics Score, Radiomics Nomogram, and Clinical Model

**Figure 6:**
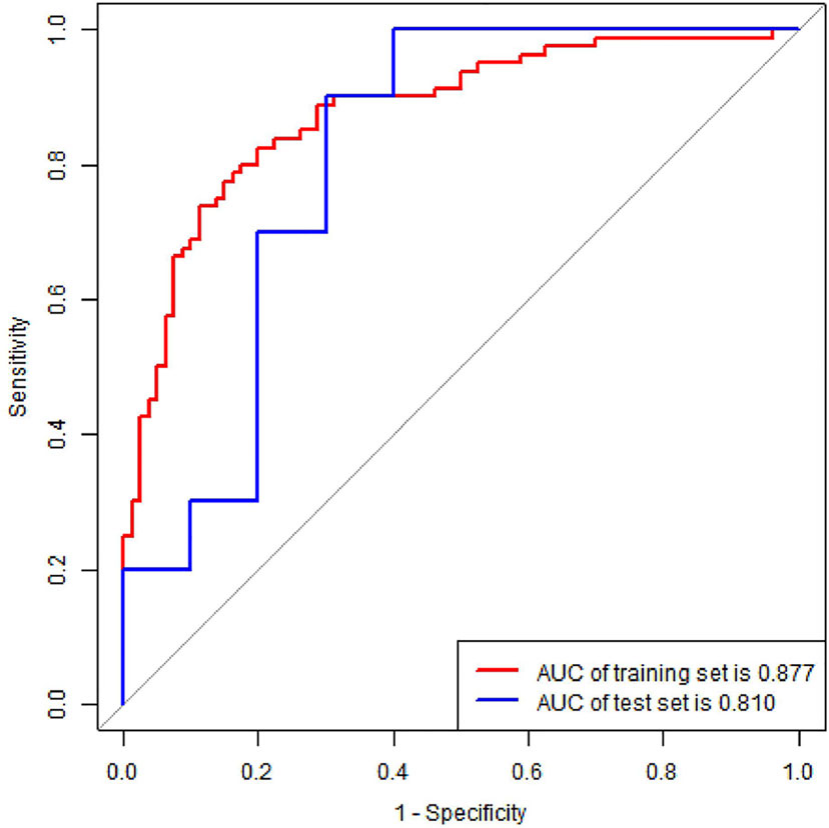
ROC curve of radiomics nomogram in the training and validation set. AUC, area under the curve.

The radiomics nomogram yielded AUC values of 0.877 (95% CI, 0.816–0.924) and 0.810 (95% CI, 0.575–0.948) in the training and validation sets, as shown **Figure 6**, respectively. The results showed that our nomogram had favorable predictive performances. The AUCs of the radiomics score and clinical model were 0.861 (95% CI, 0.798–0.911) and 0.668 (95% CI, 0.589–0.74) in the training set and 0.81 (95% CI, 0.575–0.948) and 0.55 (95% CI, 0.315–0.769) in the validation set, respectively. The diagnostic performances of three models are shown in **Table 3**. The results revealed that radiomics nomogram showed the highest accuracy, SEN, SPE, PPV, and NPV in both sets.

**Table 3:** Predictive performances of the three models.

Metrics		Accuracy	SEN	SPE	PPV	NPV
Radiomics score	Training set	0.79	0.74	0.85	0.83	0.76
	Validation set	0.80	0.90	0.70	0.75	0.88
Clinical model	Training set	0.69	0.69	0.70	0.70	0.69
	Validation set	0.65	0.70	0.60	0.64	0.67
nomogram	Training set	0.81	0.78	0.85	0.84	0.79
	Validation set	0.80	0.90	0.70	0.75	0.88

SEN, sensitive; SPE, specificity; PPV, positive predictive value; NPV, negative predictive value.

**Figure 7:**
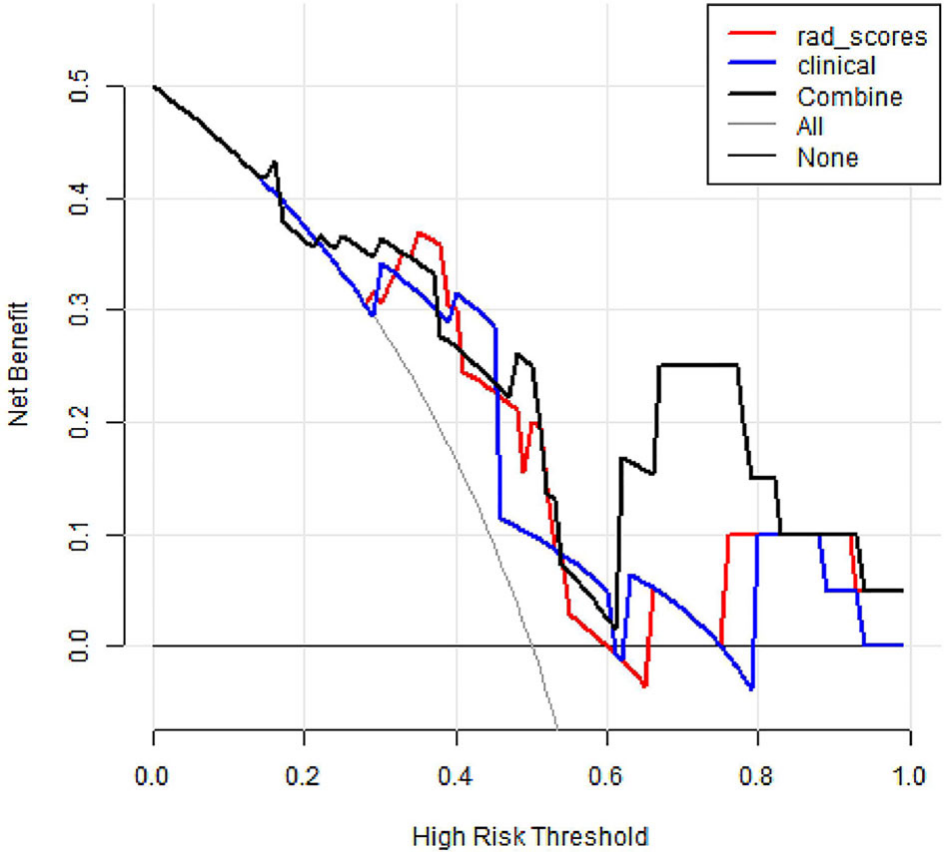
Decision curve analysis of three models. The y-axis measures the net benefit. The black thick line represents the radiomics nomogram. The red thick line represents the radiomics score. The blue thick line represents the clinical model. The gray line represents the assumption that all patients gained substantial benefit after NAC. The horizontal black thin line represents the assumption that no patients gained substantial benefit after NAC.

DCA was conducted to assess the benefit of the radiomics nomogram in **Figure 7**. The results showed that radiomics nomogram presented the greatest net benefit compared with either the treat-all patients or the treat-none patients strategies at between 0.24 to 1 threshold probability.

## Discussion

In the study, the proposed CESM-based radiomics nomogram showed a favorable pretreatment predictive ability for NAC-ineffective patients in breast cancer. Although prior studies proved that CESM seems at least as reliable as MRI in assessing response to NAC (14), the novelty of our findings may help to predict breast cancer response to NAC.

The means of predicting response to NAC was a key issue in the study. With the flourishing applications of radiomics, MRI, mammography, ultrasonography, diffuse optical spectroscopic, and positron emission tomography/computed tomography (PET/CT) have been successfully applied in treatment evaluation. For example, Antunovic et al. assessed the value of PET/CT radiomics features in predicting pathological complete response and used two approaches, namely, complete case and sensitivity, to build four radiomics models (30). Their model 4 showed the best discrimination, yielding an AUC of 0.73 without independent internal validation. Unlike the above research, we developed and validated a CESM-based radiomics model with a larger sample size (sample size, 117 vs. 79) and improved discriminatory power (AUC, 0.81 vs. 0.73). Moreover, in clinical practice, our CESM-based radiomics nomogram displayed a promising application prospect due to the mentioned strengths of CESM examination. From the results of the paper, we found that the radiomics model achieved a significantly better discriminative ability compared to clinical model (AUC, 0.81 vs. 0.55, p < 0.01). Adding clinical factors did not significantly improve the performance of the radiomics model (0.81vs. 0.81, p = 1.00), which may be caused by small sample size and unbalanced distribution of sample. We will continue our research with larger sample size in the future. The results of DCA proved that radiomics nomogram presented the greatest net benefit compared with either the treat-all patients or the treat-none patients strategies. It is worth noting that BPE was integrated into the radiomics nomogram in our study, which is consistent with recent evidence stating that BPE may be a novel predictor of treatment outcomes (31, 32). However, other studies found no significant relationship between BPE and response to NAC (33, 34). Thus, the relationship of BPE with response to NAC may be further discussed due to the variability and subjectivity inherent in the qualitative assessments of BPE. It was worth noting that most patients (84%) received taxane-based NAC schemes, according the National Comprehensive Cancer Network (NCCN) guideline (1), and 19 patients (16%) received anthracycline- and taxane-based NAC schemes. Although the distribution of treatment regimen was imbalanced, it was in accordance with the actual situation in clinical practice. Moreover, patients with different treatment regimens were divided randomly into two sets because of small sample size, which may affect the results of the study.

Our retrospective, single-institutional study exhibits several limitations. Firstly, the study includes a small sample size. The limited number of events (i.e., NAC-ineffective), related to the novelty of CESM examination and rigorous patient inclusion criteria, compromises the generalization of the results. Future work should include a highly standardized, large, multicenter dataset across patients and institutions. Secondly, ROIs were outlined manually by experienced radiologists. Although we sought to avoid inter- and intra-observer variabilities by using ICCs, this may still hinder the performance of the nomogram. Finally, compared with traditional radiomics method used in the study, the performance of the prediction model may be improved to some degree based on deep learning (DL). DL methods, such as convolution neural network, are emerging machine learning technologies suitable for to classification task, and DL application will be the priority of our future studies.

In conclusion, the proposed CESM-derived radiomics nomogram may provide a non-invasive tool for predicting response to NAC. A large sample size, multicenter, multimodal study with advanced image analysis should be further conducted to improve the performance of radiomics nomogram in predicting NAC-insensitive patients with breast cancer.

## Data Availability Statement

The original contributions presented in the study are included in the article/supplementary material. Further inquiries can be directed to the corresponding authors.

## Ethics Statement

This study was reviewed and approved by the Research Ethics Committee of Yantai Yuhuangding Hospital, and patient informed consent was waived.

## Author Contributions

HX and NM designed the study. NG, SD, and JC performed analyses. ZW and FL wrote the manuscript. HM, YS, PY, KZ, and JD collected data. ZW, FL, HX, and NM contributed to the discussion and manuscript revision. All authors contributed to the article and approved the submitted version.

## Funding

This study was supported by the National Natural ScienceFoundation of China (82001775).

## Conflict of Interest

NG, RZ, and JC were employed by the company Huiying Medical Technology Co., Ltd. SD was employed by the company GE Healthcare.

The remaining authors declare that the research was conducted in the absence of any commercial or financial relationships that could be construed as a potential conflict of interest.
